# A Weak-Magnetic-Field Measurement System with Large-Scale Uniformity and a Low Limit of Detection

**DOI:** 10.3390/nano16120719

**Published:** 2026-06-10

**Authors:** Qingzhi Meng, Yongshuai Wang, Xianfeng Liang, Yixue Wang, Yang Lu, Dengfeng Ju, Yuan Zhang, Qijing Lin

**Affiliations:** 1 State Key Laboratory for Manufacturing Systems Engineering, Xi’an Jiaotong University, Xi’an 710049, China; qzmeng2022@xjtu.edu.cn (Q.M.); yswang@stu.xjtu.edu.cn (Y.W.); 2 Department of Power Sensing, China Electric Power Research Institute Co., Ltd., Beijing 100192, China; liangxianfeng@epri.sgcc.com.cn (X.L.); wangyixue@epri.sgcc.com.cn (Y.W.); luyang1@epri.sgcc.com.cn (Y.L.); judengfeng@epri.sgcc.com.cn (D.J.); 3 Faculty of Humanities and Foreign Languages, Xi’an University of Technology, Xi’an 710054, China; zhangyuan_0212@stu.xjtu.edu.cn; 4 Faculty of Humanities and Social Sciences, Xi’an Jiaotong University, Xi’an 710049, China; 5 Shandong Laboratory of Yantai Advanced Materials and Green Manufacturing, Yantai 265503, China; 6 Xi’an Jiaotong University (Yantai) Research Institute for Intelligent Sensing Technology and System, Xi’an Jiaotong University, Xi’an 710049, China; 7 Chongqing Key Laboratory of MicroNano Systems and Intelligent Sensing, Chongqing Academician Workstation, Chongqing 2011 Collaborative Innovation Center of Micro/Nano Sensing and Intelligent Ecological Internet of Things, Chongqing Technology and Business University, Chongqing 400067, China

**Keywords:** weak magnetism, measurement system, uniform magnetic field, limit of detection

## Abstract

This paper introduces a weak-magnetic-field measurement system characterized by a large-scale uniform magnetic field and a low magnetic limit of detection (LOD). The system employs a four-ring coil assembly housed within a multi-layer magnetic shielding cavity, generating a uniform magnetic field region of 120 mm while achieving a minimum LOD of less than 10 pT. The performance of the weak-magnetic-field measurement system is appropriately validated using a bulk magnetic–electric (ME) sensor. The experimental results confirm the system’s dual functionalities in both magnetic sensor calibration and the measurement of weak magnetic parameters. Notably, this methodology is readily applicable to various forms of weak-magnetic-field measurement.

## 1. Introduction

Magnetic measurement technology plays a crucial role across various domains, including power grids [1,2,3], renewable energy sources [4,5,6], and healthcare applications [7,8,9]. To accurately measure and calibrate magnetic sensors or modules, an environment that offers a wide range, high uniformity, and minimal disturbances is imperative. The fundamental components of a magnetic measurement system include the magnetic induction coil and the magnetic shielding cavity, which are essentially utilized to generate the magnetic field and mitigate external magnetic interference, respectively. The Helmholtz coil is the most commonly utilized device for generating magnetic fields [10,11,12]. It consists of two coaxial circular coils that produce a uniform magnetic field over a specified range, and its hollow design facilitates the placement of tested devices. However, in a Helmholtz coil, the region with a highly uniform magnetic field is confined to a relatively small and central area, making it difficult to accommodate the requirements of larger-scale devices. To expand the range of uniform magnetic fields, the coil radius must be increased, but this complicates the system and elevates costs. Thus, establishing a wide range of uniform magnetic fields within a confined radius is essential. Typical magnetic sensors and modules applied in power systems are usually within 50–100 mm in size [13,14,15], so for practical applications and to save costs, a uniform magnetic field range of around 120 mm is necessary. Moreover, for detecting weak magnetic signals, a high-quality magnetic shielding environment is also required in a magnetic measurement system. Although the existing magnetic shielding systems are relatively mature, there are still shortcomings. Superconducting magnetic shielding systems [16,17,18] can achieve an equivalent magnetic noise at the 1 fT/Hz^1/2^ level, but they are limited by the physical mechanism of the superconducting ring; they can only detect low-frequency (<1 kHz) magnetic fields. The extremely small uniform magnetic field region limits their ability to detect magnetic parameters such as the magnetostriction coefficient and coercivity of thin films or materials with small dimensions. Furthermore, the requirement for low-temperature superconducting rings and complex cooling systems increases costs and operational difficulties. Active–passive shielding systems [19,20,21] can effectively compensate for DC geomagnetic and 50 Hz power frequency magnetic field interference, controlling the remanence in the magnetic shielding cavity to less than 10 nT. However, the additional compensation coil will also increase the volume and cost, and during the calibration of magnetic sensing elements, the compensation coil may subject the devices to disturbance. In a dual-planar-coil magnetic compensation system [22,23], the interaction between the coils and the shielding material distorts the magnetic field and compromises its uniformity. Magnetic field uniformity can decrease by up to 10%, especially in cases where the coupling effect is not effectively suppressed. A magnetic shielding system with a multi-layer Permalloy (Ni/Fe alloy) structure can effectively reduce magnetic noise and improve the shielding factor [24,25,26]. Due to the complexity of its structure and the way it is manufactured, precise interlayer material selection and structural design are required. However, increasing the number of layers could lead to greater mass and operational inconvenience. Therefore, the structural parameters of the Permalloy layers need to be methodically designed. Despite the widespread adoption of current weak-magnetic-field measurement systems, they remain hindered by challenges such as low resolution and considerable measurement errors.

To overcome the limitations of current weak-magnetic-field measurement systems, we propose a compact, large-volume uniform magnetic field measurement system without a complex cooling system or compensating coils. The structural parameters of the magnetic induction coil, magnetic shielding layer material, and shielding cavity were systematically designed and optimized, enabling a high uniform magnetic field and a low limit of detection (LOD). This system effectively addresses the limitations associated with the confined uniform area of current weak magnetic field measurement systems like SQUID and PPMS systems and can detect extremely weak magnetic fields for the measurement and calibration of magnetic sensors or modules on a large scale. Furthermore, this article provides a detailed introduction on how to achieve a larger uniform magnetic field region while keeping the system as compact as possible, and this insight can be extended to the development of other weak-magnetic-field measurement systems.

## 2. Theory and Simulation

### 2.1. Design of the Four-Ring Coil

**Figure 1:**
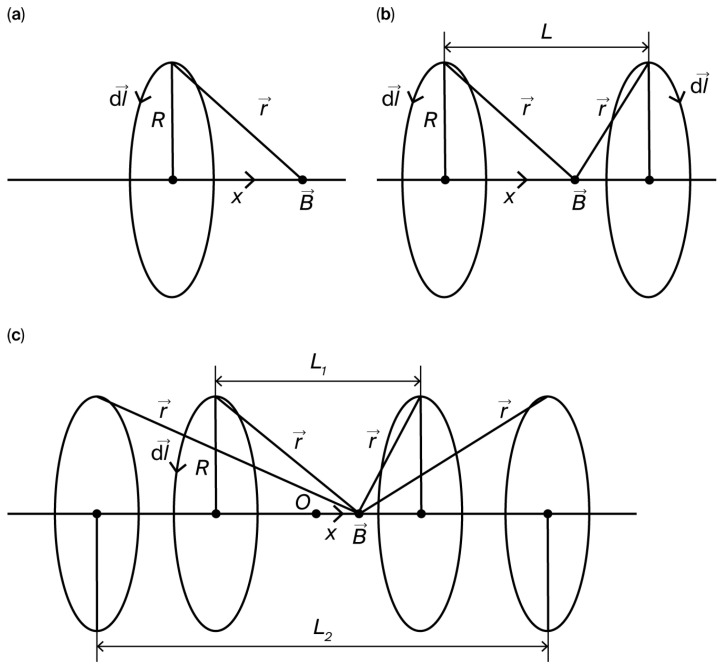
Diagram of the induced magnetic field generated by a (**a**) one-ring coil, (**b**) two-ring coil, and (**c**) four-ring coil.

The Biot–Savart law is expressed using the following formula for the contribution of a magnetic field from a current element at a point in space:

(1) $$dB=\frac{\mu_{0}}{4\pi }\frac{Id\vec{l}\times \vec{r}}{r^{3}}=\frac{\mu_{0}}{4\pi }\frac{Id\vec{l}\sin\theta }{r^{2}}$$

(2) $$\vec{B}=\int_{0}^{2\pi R}\frac{\mu_{0}}{4\pi }\frac{Id\vec{l}\times e_{r}}{r^{2}}$$

where *B* represents magnetic induction intensity, *μ*_0_ = 4π × 10^7^ A/m is the permeability of a vacuum, $Id\vec{l}$ denotes the current element vector along the coil, $\vec{r}$ is the displacement vector pointing from this element to a given point on its axis, *θ* is the angle between $\vec{r}$ and the axis, and ***e_r_*** denotes the radial unit vector. When an electric current passes through a magnetic induction coil, as depicted in Figure 1a, the field along the axis can be obtained by integrating the current elements $Id\vec{l}$ distributed along the coil’s circumference as follows:

(3) $$B=\frac{\mu_{0}R^{2}}{2{\left(R^{2}+x^{2}\right)}^{3/2}}NI$$

in which *R* represents the coil’s radius, *x* denotes the axial distance from the center of the coil, *N* is the number of turns, and *I* is the applied current. When expanding the analysis to encompass two and four rings, at a point x on the central axis, the magnetic induction is determined via the superposition of the individual contributions from each coil, which are expressed as follows:

(4) $$B=\frac{1}{2}\mu_{0}NIR^{2}\left\{{\left[R^{2}+{\left(\frac{L}{2}+x\right)}^{2}\right]}^{-3/2}+{\left[R^{2}+{\left(\frac{L}{2}-x\right)}^{2}\right]}^{-3/2}\right\}$$

(5) $$\begin{array}{c} B=\frac{1}{2}\mu_{0}IR^{2}\left\{N_{1}\left\{{\left[R^{2}+{\left(\frac{L_{1}}{2}+x\right)}^{2}\right]}^{-3/2}+\left.{\left[R^{2}+\left.{\left(\frac{L_{1}}{2}-x\right)}^{2}\right]\right.}^{-3/2}\right\}\right.\right.\\ \left.+N_{2}\left\{{\left[R^{2}+{\left(\frac{L_{2}}{2}+x\right)}^{2}\right]}^{-3/2}+{\left[R^{2}+{\left(\frac{L_{2}}{2}-x\right)}^{2}\right]}^{-3/2}\right\}\right\} \end{array}$$

where *L*, *L*_1_, and *L*_2_ represent the distance between the coils (as demonstrated in Figure 1b,c).

**Figure 2:**
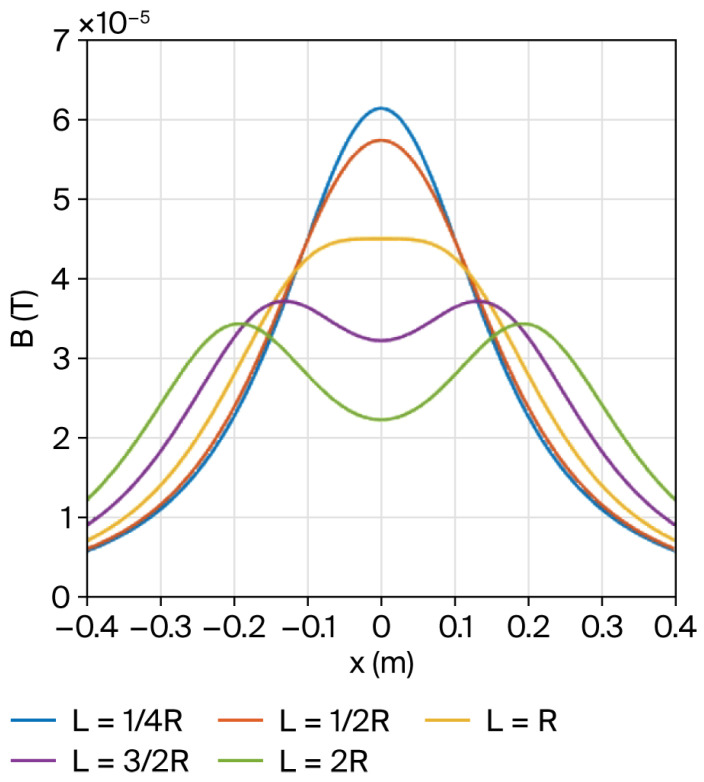
Distribution of the axial magnetic field with different coil distances (*R* = 0.1 m, *N* = 100).

To optimize the dimensions of the magnetic induction coil, based on the aforementioned model, MATLAB (R2025B, version 25.2.0.2998904) was utilized to simulate the magnetic field distribution and uniformity of the coil. Figure 2 presents the simulated magnetic field distribution along the center axis for different coil distances *L*. The analysis conducted reveals that when the degree of coil separation is shorter than the radius *R*, the magnetic field distribution is predominantly concentrated at the midpoint of the axis. Conversely, when the spacing between the coils exceeds the coil radius *R*, the magnetic field strength at the center of the axis significantly diminishes compared to that at the ends of the coils. Notably, only when the spacing is equal to the coil’s radius can it offer a relatively uniform magnetic field at the coil’s center. However, this conventional Helmholtz coil configuration inclusively generates a limited uniform range of the magnetic field. As illustrated in Figure 2, in the case of *R* = 0.1 m, the simulation results show that the uniform magnetic field distribution is mainly constrained to a 60 mm range in the axial direction. To achieve a broader area of magnetic field uniformity, additional combinations of coils need to be explored.

**Figure 3:**
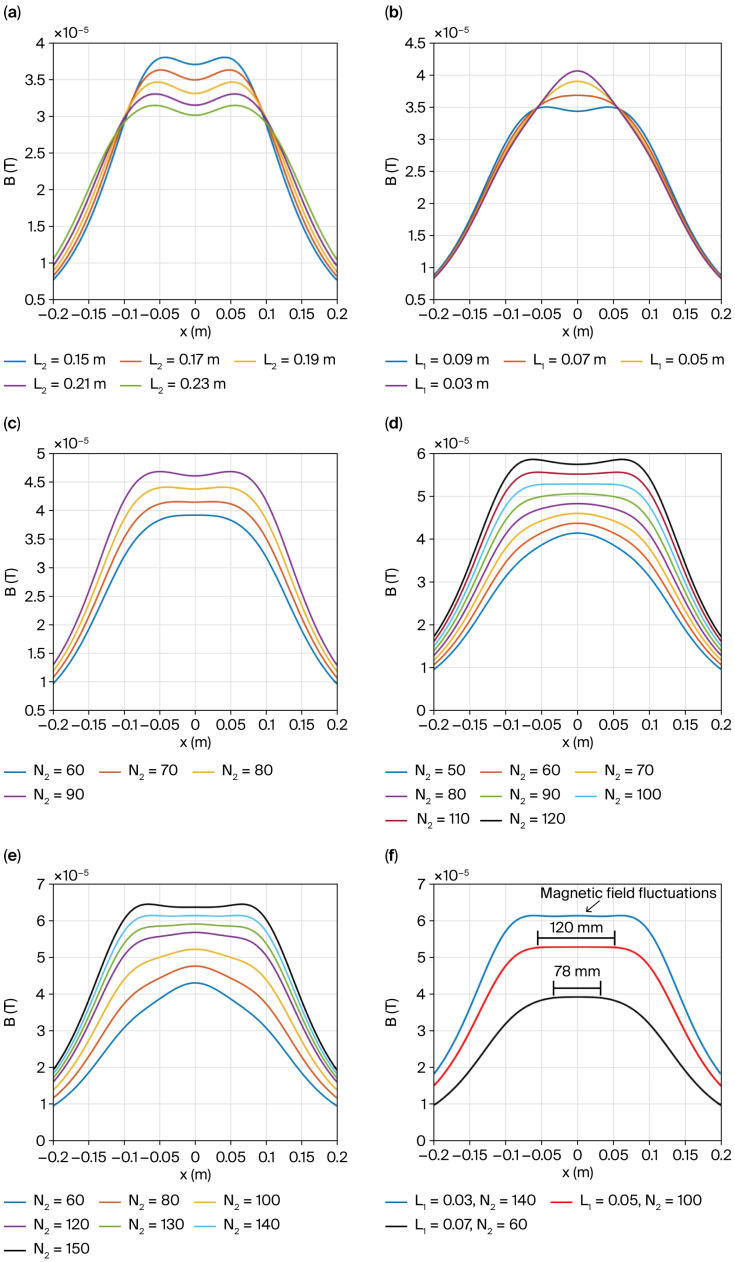
Axial magnetic field distribution for (**a**) different outer-coil distances (*L*_1_ = 0.1 m, *N*_1_ = *N*_2_ = 50); (**b**) different inner-coil distances (*L*_2_ = 0.19 m, *N*_1_ = *N*_2_ = 50); (**c**) different turns of outer coils (*L*_2_ = 0.19 m, *L*_1_ = 0.07 m); (**d**) different turns of outer coils (*L*_2_ = 0.19 m, *L*_1_ = 0.05 m); (**e**) different turns of outer coils (*L*_2_ = 0.19 m, *L*_1_ = 0.03 m); and (**f**) comparison of the distribution for three different parameters of coils (*L*_2_ = 0.19 m).

As shown in Figure 3a, the simulation results demonstrate that the distance between the outer coils *L*_2_ is the primary factor governing the size of the uniform field region in a four-ring coil. However, an excessive value of *L*_2_ can lead to a reduction in the strength of the magnetic field in the coil’s center. The two inner coils can effectively compensate for this weaker central magnetic field. Consequently, we initially fixed *L*_1_ and *N*_1_ at 0.1 m and 50, respectively, and investigated the effect of *L*_2_ variation on the magnetic field distribution, as depicted in Figure 3a. As *L*_2_ increases, the boundary of the effective magnetic field expands, while the central field intensity decreases. Aiming to strike a balance between the extent of the uniform field region and coil volume, we set *L*_2_ = 0.19 m. To compensate for the weakened field at the center, the spacing between the inner coils *L*_1_ was narrowed, with the resulting magnetic field distribution illustrated in Figure 3b. An overcompensation of the central magnetic field was observed when *L*_1_ was set to 0.07 m, 0.05 m, and 0.03 m. Without altering the extent of the effective field boundary, increasing the number of turns in the outer coil *N*_2_ can result in a region with a uniform magnetic field, as depicted in Figure 3c–e. A relatively uniform magnetic field zone can be realized when *N*_2_ is set to 60, 100, and 140 for the coil system configurations of *L*_2_ = 0.07 m, 0.05 m, and 0.03 m, respectively. In Figure 3f, the areas of uniform magnetic fields for the three different parameter settings are compared. For applications involving magnetic sensors and modules, a magnetic field uniformity of 1% is deemed sufficient; thus, we designate any area where the magnetic field exhibits a uniformity better than 1% as the uniformity zone. To this end, the magnetic field uniformity is defined as follows:

(6) $$\delta =\Delta B/B_{0}$$

where Δ*B* corresponds to the peak-to-peak variation in magnetic field strength (i.e., Δ*B = B_max_* − *B_min_*) in a specified area, and *B*_0_ denotes the average magnetic field strength. Figure 3f shows that at *L*_1_ = 0.03 m and *N*_2_ = 140, there are significant magnetic field fluctuations at the center of the coil. In contrast, at *L*_1_ = 0.05 m and *N*_2_ = 100, the coil displays an extensive and uniform magnetic field over a range of 119 mm, making it more suitable for the measurement of magnetic sensors and modules. Following additional minor parameter optimization, the final parameters were established, namely, *R* = 0.1 m, *L*_1_ = 0.047 m, *L*_2_ = 0.186 m, *N*_1_ = 46 turns, and *N*_2_ = 104 turns, with the uniform magnetic field region along the axis measuring 120 mm. Comparing the four-ring coil system to a Helmholtz coil reveals that the former yields a wider uniform magnetic field area while exhibiting a lower maximum magnetic field strength for the equivalent radius (*R*) and supply current (*I*). These results reveal that a four-ring coil designed in this way is more suitable for generating a broader region of uniform magnetic fields.

This design presumes the absence of magnetic fields exerting external interference. However, in real-world applications, the influence of the geomagnetic field and electromagnetic interference must not be overlooked; thus, constructing a magnetic shielding environment becomes imperative. To facilitate the design of a multi-layer Permalloy magnetic shielding cavity, COMSOL (version 6.2.0.290) simulation tools were employed.

### 2.2. Design of the Magnetic Shielding Cavity

**Figure 4:**
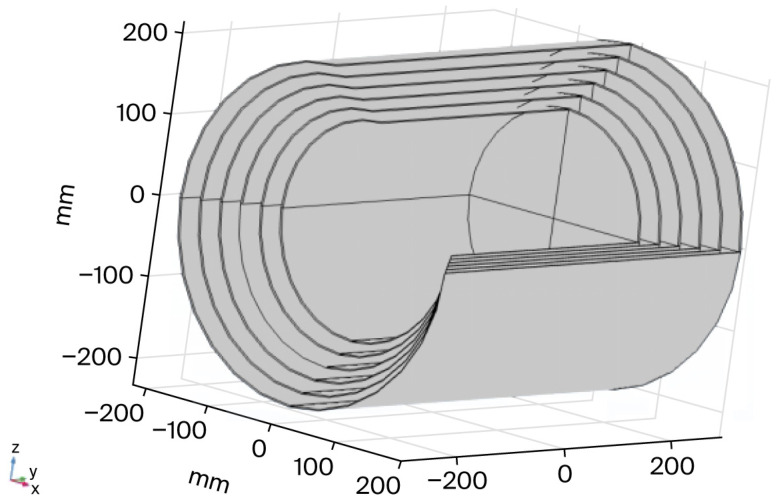
Diagram of the magnetic shielding cavity established using COMSOL.

To accommodate the dimensions (i.e., shape and size) of the four-ring coil we designed, the magnetic shielding cavity was designed as a hollow cylindrical structure, as illustrated in Figure 4. The shielding effectiveness (SE) for magnetic shielding material is expressed as a function of the impact-to-residual energy ratio:

(7) $$S_{E}=10\mathrm{lg}\frac{P_{0}}{P_{1}}=20\mathrm{lg}\frac{H_{0}}{H_{1}}$$

where *P*_0_ and *H*_0_ are the incident-plane wave power and magnetic field strength, respectively, and *P*_1_ and *H*_1_ are the corresponding values after shielding. A lower residual field after the shielding layer corresponds to higher magnetic shielding efficiency.

In order to increase magnetic shielding effectiveness, various numbers of shielding layers and their corresponding thicknesses for the shielding cavity were simulated. The primary source of interference in weak-magnetic-field measurements arises from the geomagnetic field; therefore, a 65 μT DC geomagnetic field was applied to examine the cavity’s magnetic shielding effectiveness. In our simulation, the outer shield barrel had the following dimensions: 550 mm in length, a 210 mm radius, and 1.5 mm in thickness. Permalloy was used as the shielding layer material. The lengths and radii of the other layers were reduced by 40 mm and 15 mm, respectively, for each layer. The simulation was based on the three-dimensional Maxwell’s equations, which serve as the governing model for analysis and calculation:

(8) $$\nabla \cdot E\;=\;\frac{\rho }{\varepsilon_{0}}\;(\mathrm{Gauss}’s\;\mathrm{law}\;\mathrm{for}\;\mathrm{an}\;\mathrm{electric}\;\mathrm{field})$$

(9) ∇·B = 0 (Gauss’s law for magnetism)

(10) $$\nabla \times E\;=\;-\frac{\partial B}{\partial t}\;(\mathrm{Electromagnetic}\;\mathrm{induction})$$

(11) $$\nabla \times B\;=\;\mu_{0}J\;+\;\mu_{0}\varepsilon_{0}\frac{\partial E}{\partial t}\;(\mathrm{Ampere}’s\;\mathrm{loop}\;\mathrm{law})$$

where ***E*** is electric field intensity, ***H*** is magnetic field intensity, ***B*** is magnetic flux density, ***ρ*** is electric charge density, and ***J*** is current density. The following equations were used to calculate the magnetic flux density inside the shielding layer:

(12) $$n\left(B_{1}-B_{2}\right)\;=\;\nabla_{t}\left(d_{s}B_{t}\left(H_{t}\right)\right)$$

(13) $$H_{t}\;=\;-\nabla_{t}V_{m}$$

(14) $$B_{t}\;=\;\mu_{0}\mu_{r}H_{t}$$

where ***B*****_1_** and ***B*****_2_** are the magnetic flux densities of two nodes in the layer, $\nabla_{t}$ represents a tangential derivative (gradient), *d_s_* is the layer thickness, $\mu_{r}$ is relative permeability, and *V_m_* is the magnetic scalar potential.

A magnetic insulation boundary condition was added to the innermost layer. To ensure the authenticity of the simulation results, an infinitely large air domain was added to the entire shielding barrel to simulate the working environment of the magnetic shielding cavity under realistic conditions. A coarse grid was used for this air domain to increase calculation speed, whereas a refined mesh was applied to the shielding edges to improve accuracy.

**Figure 5:**
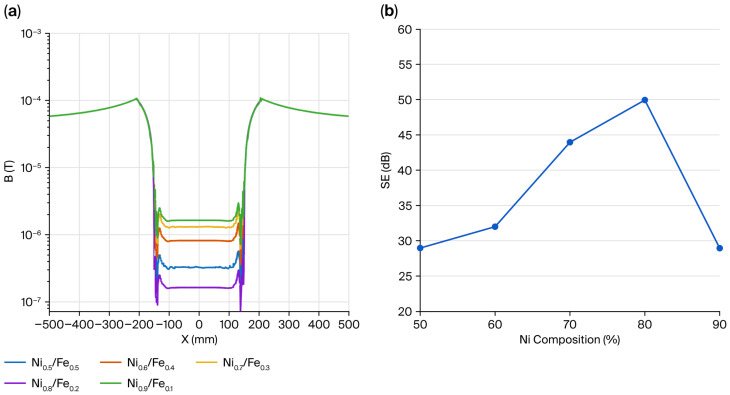
(**a**) Magnetic field distribution for different Ni compositions, inside and outside the cavity. (**b**) Shielding effectiveness (SE) versus different Ni compositions.

Figure 5a analyzes the influence of the Ni/Fe content of Permalloy on the effectiveness of magnetic shielding. Different compositions of Ni, ranging from 50% to 90%, were selected for finite-element simulation of the magnetic distribution across the shielding cavity. The strength of the magnetic field outside the magnetic shielding cavity was close to that of the geomagnetic field and dropped sharply upon entering the cavity, reaching a stable value in the cavity’s central region. The variation in shielding effectiveness with Ni/Fe content is shown in Figure 5b. As the Ni content increases, the shielding effectiveness rises first and then falls, reaching a maximum value of 49.89 dB at 80% Ni content. This is because when the Ni composition is around 80%, both the magnetocrystalline anisotropy constant *K*_1_ ≈ 0 and magnetostriction coefficient *λ* ≈ 0 are simultaneously satisfied, enabling the Permalloy material to achieve maximum magnetic permeability *μ* and minimum coercivity *H_c_*.

**Figure 6:**
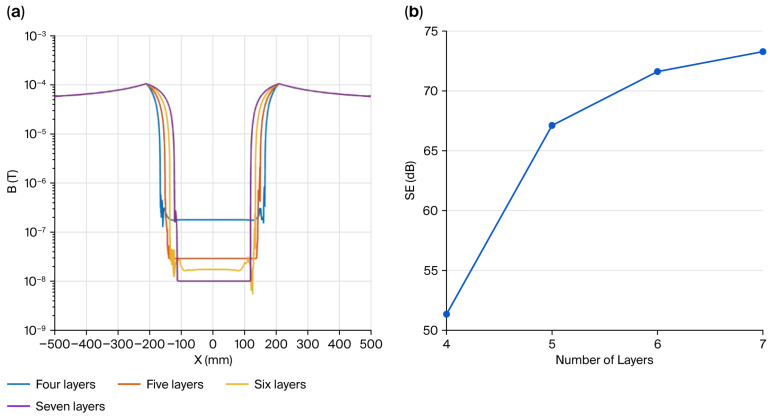
(**a**) Field distribution inside and outside the cavity for different quantities of shielding layers. (**b**) Shielding effectiveness (SE) versus the number of shielding layers.

Figure 6a illustrates the magnetic field distribution across the shielding cavity, with different quantities of shielding layers (four to seven). The findings presented in Figure 6b indicate that while there is a positive correlation between the number of shielding layers and shielding effectiveness, the benefits tend to diminish with an increase in the number of shielding layers. This phenomenon is essentially due to the intrinsic nonlinear permeability of Permalloy, wherein the inner layers experience significantly reduced field strength relative to the outer layers. This reduction leads to a decrease in both magnetic permeability and overall magnetic shielding effectiveness. Furthermore, additional shielding layers are associated with increased material costs, greater system volume, and heightened assembly complexity. After comprehensive analysis, a six-layer configuration emerged as the optimal design choice.

**Figure 7:**
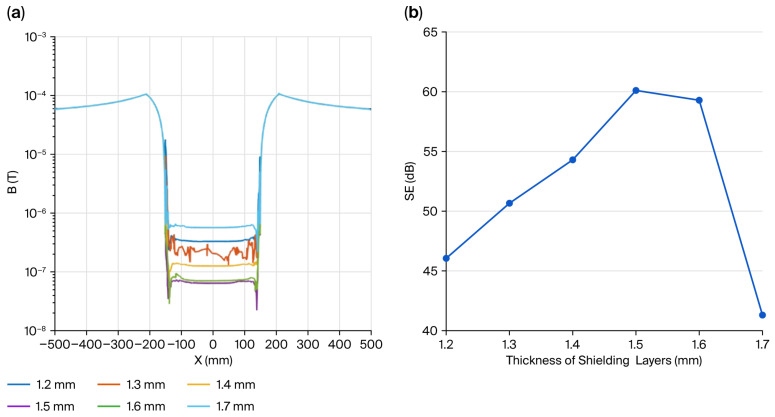
(**a**) Magnetic field distribution for different shielding layer thicknesses inside and outside the cavity. (**b**) Shielding effectiveness (SE) versus number of shielding layers.

With the internal and external diameters of the cavity held constant, Figure 7a,b show SE with respect to shielding layer thickness. The results indicate an initial increase in shielding effectiveness when layers are thicker, followed by a decline. Specifically, when the thickness is below 1.5 mm, the magnetic shielding layer demonstrates a relatively high degree of magnetic saturation, adversely affecting SE. Consequently, while increasing the thickness of the shielding layer generally improves shielding effectiveness, excessive thickness reduces the air gap between layers, thereby enhancing magnetic coupling of layers and weakening the multi-layer magnetic shielding effect. Additionally, in practical scenarios, thicker magnetic shielding layers may introduce stress or lattice defects during fabrication, consequently reducing effective magnetic permeability and reducing magnetic shielding effectiveness. Based on this evaluation, an optimal thickness of 1.5 mm is recommended for the shielding layers.

## 3. Experimental Results and Discussion

**Figure 8:**
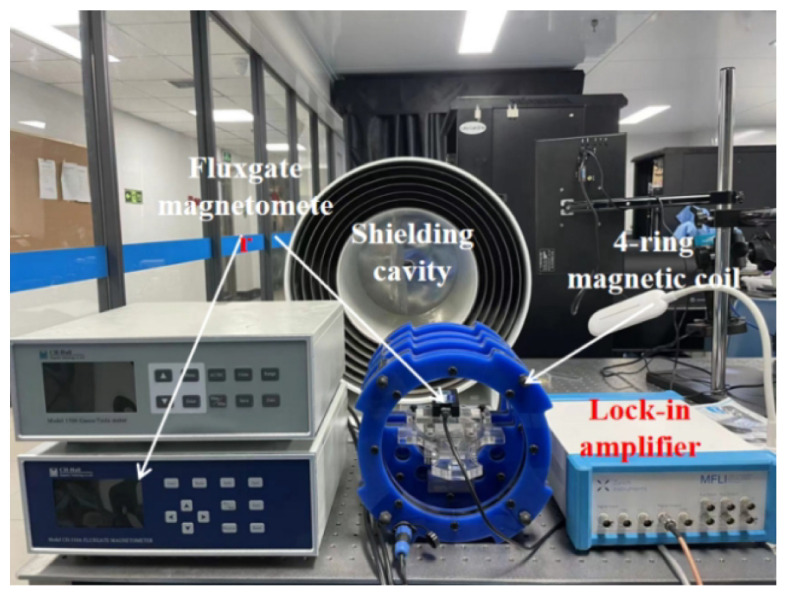
The established weak-magnetic-field measurement system.

We constructed a weak magnetic field measurement system incorporating a four-ring magnetic coil and a magnetic shielding cavity based on the designed structural parameters, as illustrated in Figure 8. The nested four-ring magnetic coil within the magnetic shielding cavity can generate a broad spectrum of uniform magnetic fields. Additionally, the multi-layer magnetic shielding cavity significantly mitigates interference from the geomagnetic and environmental magnetic fields, thereby ensuring that the four-ring can produce extremely weak magnetic fields with minor fluctuations. For the four-ring coil process, first, a frame was constructed according to the designed dimensions. Then, a winding machine was used to wrap copper wires around it in circles. Finally, the coil was covered with insulating paint and a protective shell. The shielding layer material was made of Permalloy with an 80%Ni content, selected according to the simulation design. The layer was processed into a cylindrical ring shape through cutting and rounding techniques and then subjected to annealing at 1100 degrees to eliminate the stress generated during preparation. Finally, the shielding layers were assembled without any gaps, and a stainless-steel shell was added for protection.

A lock-in amplifier (MFLI-5M, Zurich Instruments, Zurich, Switzerland) supplied DC/AC power to the coil and facilitated the readout of electrical signals. A magnetic induction coil sensitive probe (AMS-500K, Beijing Cuihai Jiacheng Magnetic & Electric Technology Co., Ltd., Beijing, China) was used to measure the induced magnetic field intensity covering the frequency range of 2–500 kHz, while a fluxgate magnetometer (Mag-670, Bartington Instruments Ltd., Witney, UK) was utilized for measurements within the DC-1 kHz range.

For practical measurement, the sample was placed or stuck onto a platform of the four-ring coil, and the fluxgate magnetometer/sensitive probe for magnetic field calibration was plugged into the slot under the platform. Then, the coil, along with the sample, was placed into the magnetic shielding cavity. If the sample needed to be connected to an external testing instrument, the wire could be fed out through the wire hole on the lid of the shielding cavity.

**Figure 9:**
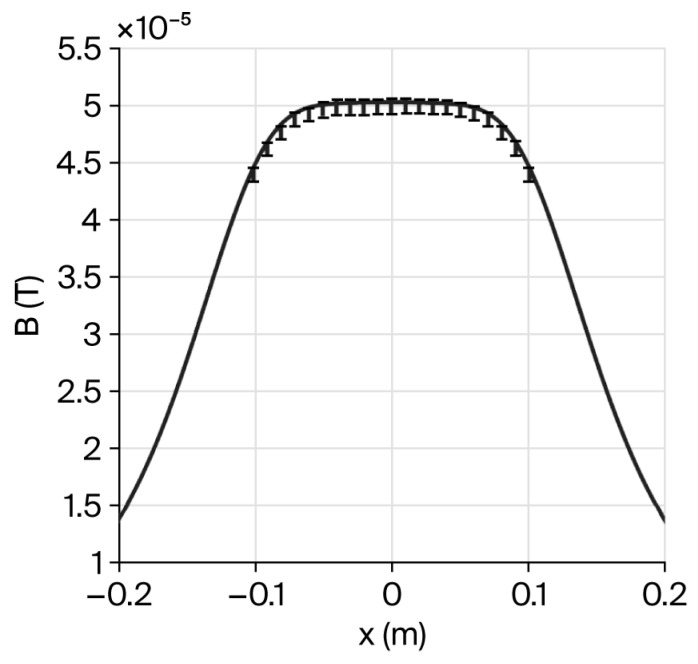
Comparison between simulation and experimental results regarding magnetic field distribution uniformity.

The performance of the weak-magnetic-field measurement system was experimentally tested, with the results compared against simulation data. The magnetic field distribution of the four-ring coil was characterized via the fluxgate magnetometer (Mag-670). To maximize shielding effectiveness against the geomagnetic field, the axis of the magnetic shielding cavity was aligned in the east–west direction, and the probe was aligned with the central axis of the coil, pointing eastward. Magnetic field intensity was measured along the coil’s axis at 2 mm intervals from one end to the other, as represented in Figure 9. A highly uniform magnetic field was observed in the central area of the coil. The calculated 120 mm region of high field uniformity from Equation (6) closely matches the simulation results. There is a minor deviation between the experimental and simulation results because the direction of the magnetic-sensitive probe may not be completely parallel to the coil axis, resulting in a slight decrease in the actually measured axial magnetic field.

**Figure 10:**
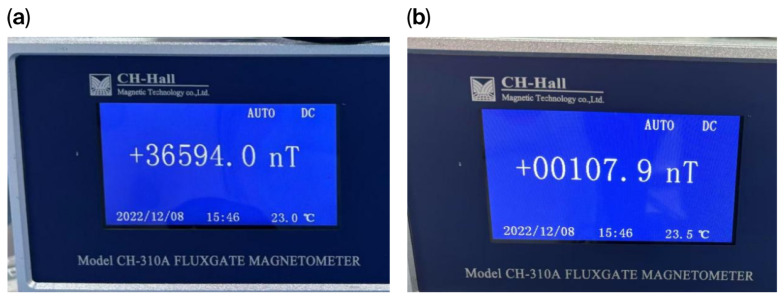
DC magnetic field intensity (**a**) outside the magnetic shielding cavity and (**b**) inside the magnetic shielding cavity.

Magnetic shielding effectiveness (*S_E_*) was computed using Equation (7) by measuring the geomagnetic field both outside and inside the magnetic shielding cavity using the fluxgate magnetometer. This magnetometer was positioned along the axis of the four-ring coil for geomagnetic measurements inside and outside the shielding cavity. The geomagnetic fields obtained before and after magnetic shielding are shown in Figure 10a,b. The uncertainty of the fluxgate magnetometer is ±2.1 nT when the field strength is between 100 nT and 1000 nT (*k* = 2) and ±31.4 nT (*k* = 2) when the field strength is between 30,000 nT and 40,000 nT. All experimental data were obtained by taking three measurements and taking the average to ensure the results were accurate. The magnetic shielding effectiveness calculated based on Equation (7) was 56.63 dB, which is 5.81% lower than the simulation result. As the magnetic shielding material exerts a hysteresis effect, after the material is magnetized, its internal magnetic field will not return to zero when the external magnetic field disappears. In addition, the lattice defects and internal stress in magnetic shielding materials act as pinning points for the magnetic domain motion and discrepancies between the actual magnetic permeability and simulation model, leading to local remanence enhancement. There are also other factors to consider, such as leakage at the cap of the shielding cavity or the magnetic sensing element not being perfectly centered on the coil axis. Because the output of magnetic sensors is highly sensitive to the field’s direction and angle, even a slight tilt of the coil can alter the readings. These factors lead to the presence of tiny residual magnetic fields that cannot be totally eliminated, thereby resulting in a minor deviation from the simulation results. Nevertheless, the SE is sufficiently high to facilitate measurement of weak magnetic fields within the system.

**Figure 11:**
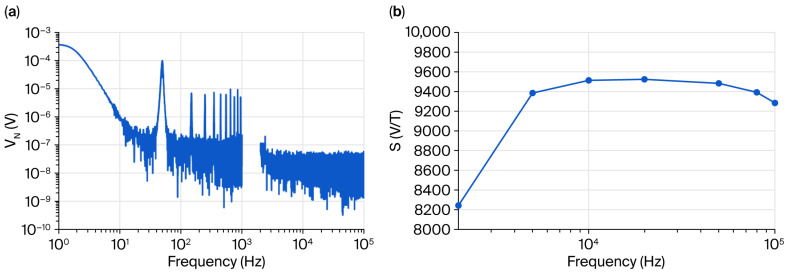
(**a**) The background noise of the system from 1 Hz to 1 kHz and 2 to 100 kHz. (**b**) A sensitivity curve of the detection probe from 2 to 100 kHz.

The system’s capacity for weak-magnetic-field signal detection was evaluated by assessing its limit of detection (LOD). The magnetically sensitive probe (ASM-500k) was positioned in the uniform area of the four-ring coil. The supplied voltage was adjusted to an amplitude at which the response voltage just exceeded the background noise of the system, thus allowing for the determination of the LOD:

(15) $$LOD\;=\;\frac{V_{N}}{S}$$

where *V_N_* represents background noise voltage, and *S* denotes the sensitivity of the magnetic sensor. For practical applications, *V_N_* should be taken to be a response voltage slightly higher than the background noise. All tests were performed under controlled conditions, with a temperature of 24 °C (297.15 K) and 72% relative humidity. The measured *V_N_* of the system across the frequencies of 1 Hz to 1 kHz and 2 kHz to 100 kHz is depicted in Figure 11a. The sensitivity of the fluxgate magnetometer (Mag-670) within the 1 Hz to 1 kHz range is 10^4^ V/T, and the sensitivity of the magnetic-sensitive probe (ASM-500k) from 2 kHz to 100 kHz is illustrated in Figure 11b. Since the ASM500k has a reading uncertainty of ±5% within the 2–100 kHz range, it is necessary to perform three scans and average the data to calculate the LOD. By integrating the data presented in Figure 10a,b with Equation (15), the LOD of the system can be determined. At a low frequency of 1 Hz, the LOD is 35.3 nT, and in the frequency range of 1–20 Hz, the LOD drops as the frequency increases. Between 20 and 40 Hz, the LOD is in the order of 40 pT, while in the frequency range of 40–60 Hz, influenced by the 50 Hz power frequency signals, both the background noise and LOD of the system increase significantly, especially at a power frequency of 50 Hz and its harmonic frequencies below 1 kHz, where the LOD is within 10 nT to 1 nT. As the frequencies increase to 2–100 kHz, the LOD decreases to 10 pT. This sensitivity surpasses that of most currently available magnetic sensors, indicating that the proposed system is proficient in calibrating the performance of magnetic sensors, even in extremely weak-magnetic-field conditions.

The instability of the LOD is mainly due to background noise in the environment, electromagnetic noise from devices used in the measurement system, and noise contributed by the fluxgate magnetometer and the magnetic-sensitive probe. The background noise in the environment includes disturbance from the geomagnetic field as well as the subway and automobiles outside the lab. This noise was reduced by designing and employing high-performance magnetic shielding structures, as discussed in Section 2.2. The electromagnetic noise is mainly caused by the operation of instruments at a power frequency of 50 Hz, which generates a magnetic field that radiates into space, contributing to noise fluctuations, especially at low frequencies. To reduce the interference from the power frequency, a notch circuit for 50 Hz and its harmonic frequencies is usually added to the front of the device being measured, and it is recommended to avoid testing sensor performance at or around this frequency. The noise from the fluxgate magnetometer and the magnetic-sensitive probe consists of thermal and 1/*f* noise. As this type of noise is inherent to sensitive components, it can only be suppressed by adjusting the parameters of the lock-in amplifier during measurement. When weak signals are being detected, the measurement bandwidth of the lock-in amplifier determines the noise suppression capacity, which is expressed as follows:

(16) $$B_{L}\;=\;\frac{FO}{2\pi TC}$$

where *B_L_* and *FO* are the bandwidth and order of the low-pass filter, and TC is the time constant. The lower bandwidth and higher order will lead to less fluctuation in the noise measured. However, the time constant increases correspondingly, which reduces testing efficiency. Hence, the *B_L_* and *FO* are fixed according to the detected frequency. When we measure the signal of the 1 Hz–1 kHz frequency, the noise fluctuation is large, and the *B_L_* and *FO* are set to 1 Hz and 6, respectively. As the noise fluctuation in the 2 kHz–100 kHz range is relatively small, the *B_L_* and *FO* are set to 10 Hz and 4, respectively.

**Figure 12:**
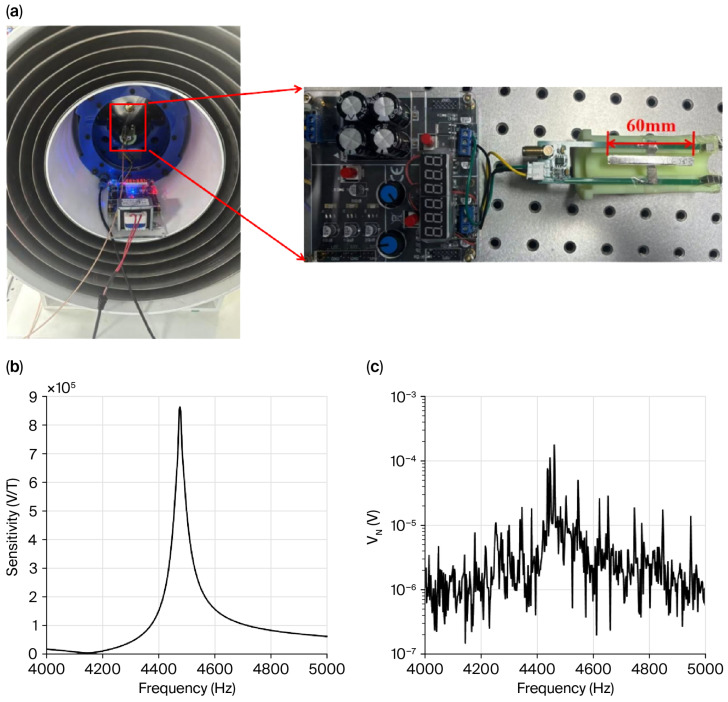
(**a**) The ME sensor being tested in the coil. (**b**) Frequency response characteristics of the ME sensor. (**c**) The background noise of the ME sensor around the resonant frequency, 4.48 kHz.

The performance of the magnetic measurement system was assessed using a bulk ME sensor. The sensor’s sensitive area is a rectangular cantilever beam measuring 60 mm × 5 mm × 1.5 mm that fits entirely within the uniform region of the coil (120 mm along the axis), as shown in Figure 12a. By keeping the supply voltage of the coil at 1 V, we obtained a resonant frequency of 4.48 kHz for the ME sensor, as presented in Figure 12b. Subsequently, the coil bias voltage was held constant at 1 V, and the magnetic probe was positioned within the coil’s uniform field region. Under these conditions, a magnetic field intensity of 1.21 μT was obtained. The sensitivity of the ME sensor was calculated as follows:

(17) $$S\;=\;\frac{V_{R}}{H}$$

where *V_R_* represents the response voltage, and *H* denotes magnetic field intensity. The sensitivity of the ME sensor was determined to be 8.65 × 10^5^ V/T@4.48 kHz. The background noise of the ME sensor is illustrated in Figure 12c. The background noise around the resonant frequency of the ME sensor varies between 1 and 100 μV, with a minimum LOD of 13.87 pT observed at this resonant frequency. These results reveal that the LOD of our measurement system is superior to that of the ME sensor, suggesting that the developed system can meet the weak magnetic requirements necessary for testing ME sensors.

Table 1 summarizes a comparison between our weak-magnetic-field measurement system, the latest approaches in the literature, and commercial magnetic measurement systems in terms of LOD, frequency range, uniform magnetic field region, and magnetic uniformity. Our weak-magnetic-field measurement system demonstrates exceptional performance in terms of LOD and wide detection frequency range. Even at low frequencies <1 kHz (excluding a power frequency of 50 Hz and its harmonics), the LOD remains below 30 pT, which is significantly lower than that of current measurement systems and most types of magnetic sensors. Notably, this performance was achieved within a large uniform region of 120 mm, which is 1.5–6 times larger than typical shielded fluxgate systems (Refs. [27,28,29]) and even exceeds the active-shielding cavity reported in Ref. [30]. Unlike commercial moment-measuring systems such as SQUIDs [31] and the Physical Property Measurement System (PPMS) [32], which mainly focus on measuring weak magnetic moment (M-moment) and multi-physics magnetic parameters, we established a compact weak-magnetic-field environment without complex cryogenic equipment for the measurement of magnetic sensors and modules. Even though these systems exhibit extremely high M-moment sensitivity and magnetic uniformity, the small chamber volume restricts their application to magnetic parameters of small materials.

These results suggest that our measurement system is well-suited to calibrating the performance of weak-magnetic-field sensors, indicating that it could be applied to areas such as magnetic bio-detection or magnetic-free field spaces. Furthermore, in terms of magnetic uniformity, the four-ring coil within our system offers an expanded uniform zone and enhanced uniformity, making it suitable for the magnetic parameter measurement of larger sensors or modules.

## 4. Conclusions

A weak-magnetic-field measurement system was designed and constructed, featuring an expanded uniform-field volume and an ultra-low LOD. A composite four-loop magnetic induction coil structure was developed with dimensional optimization to generate a highly homogeneous field, achieving a 120 mm uniformity span with 99% homogeneity. COMSOL-based modeling was used to optimize the layer number and thickness of a Permalloy magnetic shield. The finalized configuration of six shielding layers with a 1.5 mm Permalloy layer delivered a shielding effectiveness of 56.64 dB. The measurement system constructed to these specifications exhibits a minimum detectable field of 10 pT and a 120 mm uniformity region, in close agreement with the results of a simulation. Validation using a fabricated bulk ME sensor confirmed the feasibility of the proposed shielding system for weak-field measurement.

## Figures and Tables

**Table 1:** Comparison of the performance parameters of weak magnetic measurement systems.

LOD/ M-Moment Sensitivity	Frequency Range	Uniform Magnetic Field Region	Magnetic Uniformity	References
625 pT	DC/50 Hz harmonic	30 mm	98%	[27]
10 pT	DC	20 mm	99.8%	[28]
420 pT @ 380 Hz	DC-1 kHz	80 mm	\	[30]
10–15 nT	DC-100 Hz	75 mm	99%	[29]
1 × 10^−8^ emu	0.1 Hz–1 kHz (For AC magnetization)	40 mm	99.99%	[31]
10^−6^–10^−7^ emu	10 Hz–10 kHz (For AC magnetization)	55 mm	99.99%	[32]
<10 pT @ 2–100 kHz; 20 pT @ 100 Hz–1 kHz	DC-1 kHz & 2–100 kHz	120 mm	99%	This work

## Data Availability

All data are contained within this article.
